# Noninvasive intracranial pressure waveforms for estimation of intracranial hypertension and outcome prediction in acute brain-injured patients

**DOI:** 10.1007/s10877-022-00941-y

**Published:** 2022-11-18

**Authors:** Sérgio Brasil, Gustavo Frigieri, Fabio Silvio Taccone, Chiara Robba, Davi Jorge Fontoura Solla, Ricardo de Carvalho Nogueira, Marcia Harumy Yoshikawa, Manoel Jacobsen Teixeira, Luiz Marcelo Sá Malbouisson, Wellingson Silva Paiva

**Affiliations:** 1 Division of Neurosurgery, Department of Neurology, School of Medicine, University of São Paulo, 255 Enéas Aguiar Street, São Paulo, 05403000 Brazil; 2 Department of Intensive Care, School of Medicine, University of São Paulo, São Paulo, Brazil; 3 Medical Investigation Laboratory 62, School of Medicine, University of São Paulo, São Paulo, Brazil; 4 Department of Intensive Care, Erasme Hôpital, Université Libre de Bruxelles, Bruxelles, Belgium; 5 Department of Intensive Care, Universitá degli Studi di Genoa, Genoa, Italy

**Keywords:** Intracranial compliance, Intracranial pressure waveform, Intracranial hypertension, Acute brain injury, Neuromonitoring

## Abstract

Analysis of intracranial pressure waveforms (ICPW) provides information on intracranial compliance. We aimed to assess the correlation between noninvasive ICPW (NICPW) and invasively measured intracranial pressure (ICP) and to assess the NICPW prognostic value in this population. In this cohort, acute brain-injured (ABI) patients were included within 5 days from admission in six Intensive Care Units. Mean ICP (mICP) values and the P2/P1 ratio derived from NICPW were analyzed and correlated with outcome, which was defined as: (a) early death (ED); survivors on spontaneous breathing (SB) or survivors on mechanical ventilation (MV) at 7 days from inclusion. Intracranial hypertension (IHT) was defined by ICP > 20 mmHg. A total of 72 patients were included (mean age 39, 68% TBI). mICP and P2/P1 values were significantly correlated (r = 0.49, p < 0.001). P2/P1 ratio was significantly higher in patients with IHT and had an area under the receiving operator curve (AUROC) to predict IHT of 0.88 (95% CI 0.78–0.98). mICP and P2/P1 ratio was also significantly higher for ED group (n = 10) than the other groups. The AUROC of P2/P1 to predict ED was 0.71 [95% CI 0.53–0.87], and the threshold P2/P1 > 1.2 showed a sensitivity of 60% [95% CI 31–83%] and a specificity of 69% [95% CI 57–79%]. Similar results were observed when decompressive craniectomy patients were excluded. In this study, P2/P1 derived from noninvasive ICPW assessment was well correlated with IHT. This information seems to be as associated with ABI patients outcomes as ICP.

Trial registration: NCT03144219, Registered 01 May 2017 Retrospectively registered, https://www.clinicaltrials.gov/ct2/show/NCT03144219.

**Supplementary Information:**

The online version contains supplementary material available at 10.1007/s10877-022-00941-y.

## Introduction

Critically ill patients with primary brain injury or with cerebral damage secondary to extra-cerebral diseases (i.e. acute liver failure, drug intoxication) are at risk of developing intracranial hypertension (IHT) [1], a life-threatening condition that, unless an invasive catheter to measure intracranial pressure (ICP) is placed for monitoring, may elapse unnoticed [2, 3]. Nevertheless, ICP management based exclusively on predefined thresholds may ignore the complexity of intracranial compliance (ICC) [4, 5], which is influenced by several intracranial (i.e. blood flow, cerebrospinal fluid, mass effect and brain parenchyma) and systemic (i.e. carbon dioxide, sodium, temperature…) variables that result in moving ICP tolerance between different patients [6, 7]. Furthermore, other factors, such as age, cerebral autoregulation, compensatory reserve capacity and speed of IHT occurrence also contribute to the potential detrimental effects of IHT on brain oxygenation and function, pointing to the need of individualized ICP assessments rather than static thresholds to be applied to all patients [5].

Because of the invasive nature of ICP measurement techniques, many other non-invasive methods have emerged in recent years as surrogates or “triage” tools for ICP estimation, such as transcranial Doppler [8], optic nerve sheath diameter ultrasound [9], optic nerve elastography [10], tympanometry [11] and automated pupillometry [12], almost all of them having an acceptable accuracy to detect IHT and potentially guide for cerebral perfusion pressure evaluation [8, 13–15].

In this setting, a well-studied but under valuated phenomenon in clinical practice is the ICP waveform (ICPW) [13, 16]. ICPW variations have been demonstrated according to changes in intracranial volume and pressure [5, 17], indicating the possibility of this parameter to play a role in acute brain injuries. Recently, a noninvasive technique that assess micrometric skull deformations throughout the cardiac cycle and able to reproduce the ICPW (NICPW), was developed with strong correlation with invasive ICP wave morphology [18, 19], with its prognostic value still to be proven. Therefore, the objectives of this study were to assess the correlation between invasive ICP mean values and NICPW parameters, and to assess the prognostic value of NICPW parameters in acute brain-injured (ABI) patients.

## Methods

### Study design and population

This single center, cross-sectional study was conducted in six intensive care units (ICUs) of the Hospital das Clínicas, São Paulo University, Brazil between 2020 and 2022. Therefore, the present manuscript is a retrospective analysis of a prospective trial. The clinical trial study protocol was approved by the local Ethics Committee in April 2017 (Number NCT03144219, available at clinicaltrials.gov). Informed consent was obtained from legally authorized representatives/next of kin of patients before inclusion. This study was performed according to the Standards for Reporting of Diagnostic Accuracy Studies (STARD) (Supplemental Table 1).

Patients were eligible for this study if they had: acute traumatic (TBI) or non-traumatic brain injury; (b) need for ventilatory support at enrollment; (c) required invasive ICP monitoring, according to the guidelines for high-risk of brain herniation of the Brain Trauma Foundation. Exclusion criterion was the presence of fixed mydriatic or middle-sized pupils for more than 2 h after ventilatory and hemodynamic stabilization. As part of a greater study for the assessment of ICP variations over cerebral blood flow and ICP waveforms, the present analysis comprised a single 10-min session (i.e., at least 700 heart beats) for each patient, including them within the first 5 days after admission. In this model, no comprehensive variations of the recorded parameters were induced. Simultaneous recording of invasive arterial blood pressure, ICP, NICPW (see below), electrocardiogram, temperature and oxygen saturation was obtained, as previously reported [6, 19]. Data analysis was dedicated to registering ICP values and NICPW parameters, correlating them with early outcomes described in detail below.

### Neuromonitoring

ICP was measured using the Neurovent monitoring system via optic-fiber transducer (Raumedic, Munchberg, Germany); mean ICP value (mICP) over the 10-min recording was therefore calculated. NICPW were assessed using the B4C (B4C; Brain4care Corp., São Carlos, Brazil) sensor, which consisted into a monitor that quantifies local cranial bone deformations using specific sensors [20]. Physics, engineering and implementation of this system in clinical practice have been described elsewhere [19, 20] (Supplemental Fig. 1). The system was positioned in the frontotemporal region, approximately 3 cm over the first third of the orbitomeatal line, at the same side of ICP catheter implantation. Consequently, the main branches of the temporal superficial artery and the temporal muscle were avoided, and sensor contact was provided through an area of thin skin and skull bone, whereas slight pressure was applied to the adjustable band until an optimal signal was detected. The obtained waveform was equivalent to the ICP waveform obtained through invasive techniques, such as intraparenchymal probes or external ventricular derivation as observed in previous studies with smaller samples than the present [18, 19]. The distinctive ICP peaks were extensively described previously, being P1 the representation of arterial ejection and P2 the tidal wave, there is the spreading of blood volume thru the brain [21].

NICPW calculations were performed from the average of the pulses within each minute of monitoring, likewise, mICP were calculated as the average of each minute of the session. The parameter of interest obtained from the analysis of NICPW was the P2/P1 ratio, that is, the ratio form dividing the amplitudes of these two peaks. As demonstrated previously, the amplitude of P2 increases with IHT [21, 22]; this ratio is based on an algorithm previously created from the synchronization of NICPW with arterial blood pressure obtained from more than hundred thousand heartbeats [20] (Supplemental Fig. 2). For this study, brain compliance index (BCI) was calculated as = mICP*P2/P1, for each patient, to observe whether the combination of mICP with NICPW would perform satisfactorily on the correlation with outcomes.

Arterial blood pressure (BP) was recorded invasively using a radial artery catheter (Leadercath Arterial polyethylene catheter 18-gauge, 10 cm length, 0.8 mm internal diameter, 1.2 mm external diameter, Vygon, Ecouen, France), connected to a disposable pressure transducer (VAMP Plus system; Edwards Lifesciences, Irvine, CA, USA) and to a Philips MX800 IntelliVue monitor (Philips Medical System; Best, The Netherlands).

### Data collection

The clinical variables collected were demographics, previous comorbidities, final diagnosis, the Marshall tomographic score (in the case of TBI), the modified Fisher tomographic score (in case of subarachnoid hemorrhage), neurosurgical interventions (i.e., craniotomies or craniectomies), blood gas analyses before the 10-min session and concomitant administered sedatives.

### Participants and outcome endpoints

Data obtained using the B4C sensor was not used for clinical management. The primary objective of the study was the predictive value of P2/P1 ratio for mICP > 20 mmHg (IHT). Considering that all patients were assessed in early days after ABI and hospitalization, being under sedation and mechanical ventilation, the secondary objective of the study was to verify whether the information acquired could be predictive of short-term outcomes (STO), defined as (a) early death (ED group); (b) successful mechanical ventilation weaning with spontaneous breathing (SB group) or (c) dependency from mechanical ventilation (MV group). All STO where considered in up to 7 days after study inclusion.

### Statistical analyses

The sample size was calculated for the primary objective of evaluating the NICPW accuracy to discriminate IHT. Thus, to estimate an area under curve (AUC-ROC) of 0.85, with a null hypothesis value of 0.6 and 80% power, a total of 70 patients would be required [23]. For descriptive purposes, categorical variables were presented through relative and absolute frequencies and were compared using the chi-square or Fisher’s exact test, as appropriate. Continuous variable distributions were deemed normal, as assessed by skewness, kurtosis, and graphical methods. The 3 groups (ED; SB and MV) were compared through chi-square, ANOVA and Kruskal-Wallis tests, as appropriate; *post hoc* analyses to assess for specific differences between groups was performed, accordingly. The ROC curve analysis was performed using the Johns Hopkins University tool (available at www.jrocfit.org). Differences between AUROCs were assessed using the DeLong method. As decompressive craniectomy (DC) may impact intracerebral dynamics and NICPW [6, 19], a secondary analysis excluding DC patients was also performed. Previous studies indicated for patients with craniotomies or large skull fractures absence of comprehensive changes in ICC, hence no exclusion of these patients was considered for statistical analysis [6, 19].

## Results

### Study population

Over a total of 164 patients with ICP monitoring over the study period, 89 were not included because of restrictions imposed by COVID-19 pandemics. Of the remaining 75 patients, three were excluded because of poor data quality, yielding 72 patients for the final analysis, a mean of 783 ± 92 waveforms per patient, with a total sample of 56.386 cardiac pulses for parameters extraction. The overall clinical features of the sample are presented in Table 1, stratified according to the outcome subgroups. The mean age was 39 ± 21 years, 65% were male and the majority (68%) suffered TBI. There were no baseline differences regarding age, gender, comorbidities, pathology, admission GCS or neurosurgical status among groups.

**Table 1:** Main characteristics of the study population, according to early outcome

	All (n = 72)	SB (n = 15)	MV (n = 47)	ED (n = 10)	p value
Age, years	39 ± 21	35 ± 20	39 ± 20	45 ± 24	0.510
Male gender, n (%)	47 (65%)	10 (67%)	32 (68%)	5 (50%)	0.547
Parameters					
mICP, mmHg	14 (11–19)	13 (11–19.5)	13 (10–17)	21.5 (12–27)^a,b^	0.016
mICP > 20mmHg, n (%)	13 (18%)	3 (20%)	5 (11%)	5 (50%)^a,b^	0.013
P2/P1 ratio	1.15 (0.9–1.3)	0.9 (0.8–1.25)	1.1 (0.9–1.3)	1.25 (1.2–1.5)	0.066
P2/P1 ratio > 1.2	31 (43%)	5 (33%)	19 (40%)	7 (70%)	0.159
BCI	14.5 (9.9–22.2)	13.7 (11.7–19.1)	13.9 (9.3–19.0)	27.1 (21.2–36.9)^a,b^	0.004
Mean arterial pressure, mmHg	89 ± 11	88 ± 13	90 ± 12	86 ± 10	0.577
SO_2_	99 (97–100)	99 (97.5–100)	99 (98–100)	98 (97–100)	0.971
PaCO_2_	37.3 ± 5.6	37.6 ± 6.6	37.5 ± 5.6	35.5 ± 3.5	0.562
Hemoglobin, mg/dL	9.8 ± 1.7	9.6 ± 1.7	9.9 ± 1.7	9.5 ± 2.2	0.773
Temperature, °C	36.1 ± 1.0	37.0 ± 0.7	36.0 ± 0.9	35.5 ± 0.7	0.06
Pathology					0.612
TBI	49 (68%)	10 (67%)	33 (70%)	6 (60%)	
SAH	12 (17%)	2 (13%)	6 (13%)	4 (40%)	
Ischemic Stroke	7 (10%)	2 (13%)	6 (13%)	0 (0%)	
Hemorrhagic stroke	3 (4%)	1 (7%)	2 (4%)	0 (0%)	
Brain neoplasm	1 (1%)	0 (0%)	1 (2%)	0 (0%)	
Neurosurgery					0.744
No	17 (24%)	4 (27%)	10 (21%)	3 (30%)	
Craniotomy	32 (47%)	8 (53%)	23 (49%)	3 (30%)	
Craniectomy	21 (29%)	3 (20%)	14 (30%)	4 (40%)	
Mean arterial pressure, mmHg	89 ± 12	88 ± 11	90 ± 12	86 ± 10	
Comorbidities, n (%)					0.192
None	47 (65%)	8 (53%)	32 (68%)	7 (70%)	
Metabolic syndrome	20 (28%)	4 (27%)	14 (30%)	2 (20%)	
Others	5 (7%)	3 (20%)	1 (2%)	1 (10%)	
SAPS-3 score	58 ± 11	54 ± 10	58 ± 11	64 ± 13	0.082
Admission GCS	3 (3–8)	3 (3–10)	3 (3–8)	3 (3–3)	0.738

Data presented as mean ± standard deviation, median (quartiles) or counts (%). *SB* spontaneous breathing, *MV* mechanical ventilation, *ED* early death, *mICP* mean intracranial pressure, *BCI* brain compliance index, *TBI* traumatic brain injury, *SAH* subarachnoid hemorrhage, *SAPS-3* simplified acute physiologic score 3, *GCS* Glasgow coma score. P values represent statistical significance between ED and the other groups

^a^p < 0.05 vs. SB, ^b^p < 0.05 vs. MV in the post hoc analysis

### ICPW and ICP

**Fig. 1:**
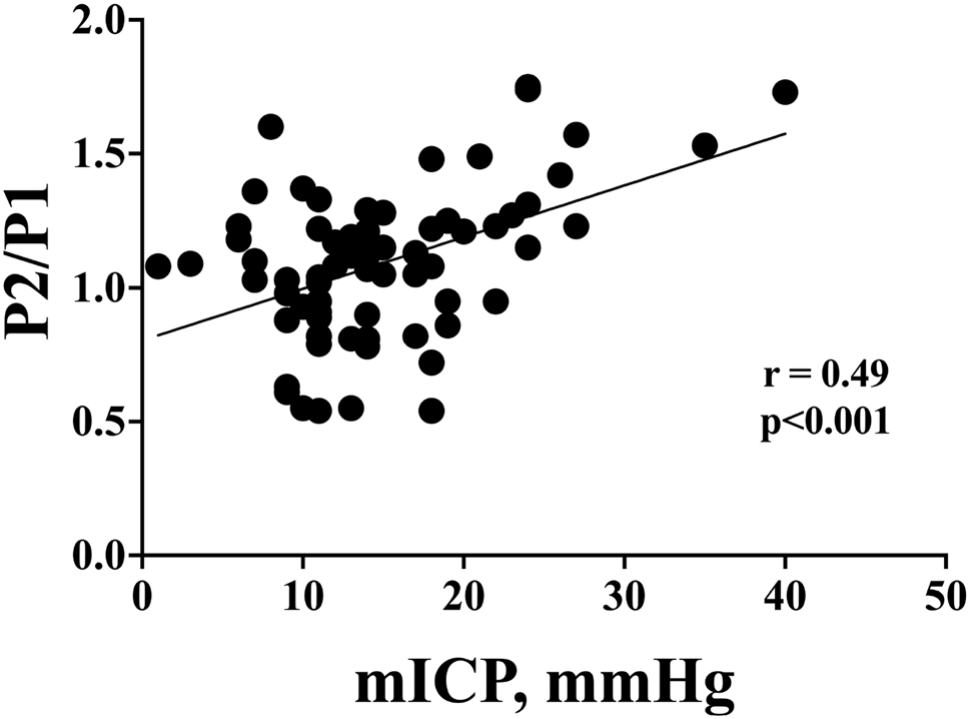
Linear correlation between mean intracranial pressure (mICP) values and P2/P1 ratio

**Fig. 2:**
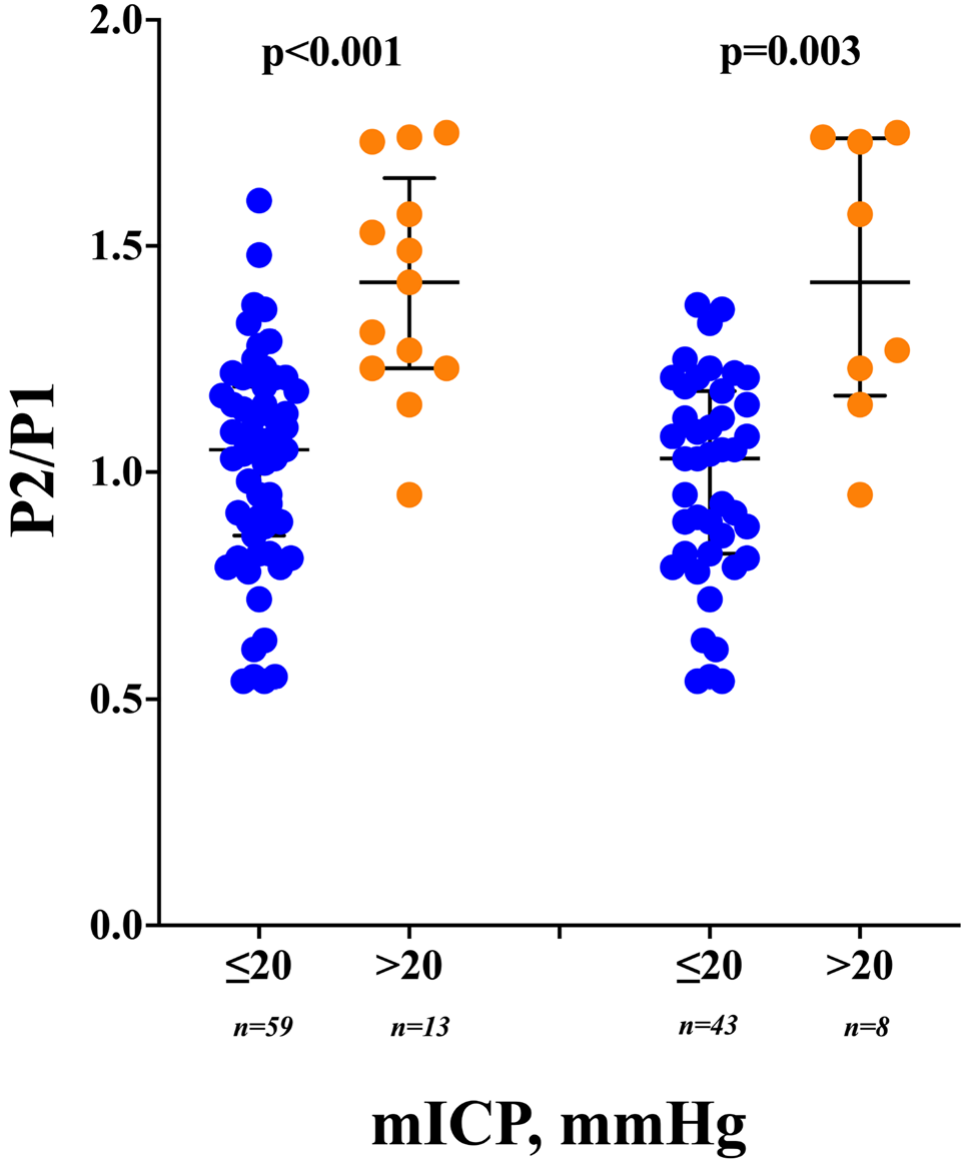
P2/P1 values according to mean intracranial pressure (mICP) values, in all patients (n = 72, left side) or after exclusion of those with decompressive craniectomy (n = 51, right side)

Overall mICP values and NICPW parameters are shown in Table 1). There was a significant correlation between mICP and P2/P1 (r = 0.49, p < 0.001 Fig. 1). P2/P1 was significantly higher in patients with IHT (Fig. 2); P2/P1 had an AUROC to predict IHT of 0.88 [95% CI 0.78–0.98], whereas the P2/P1 cut-off of > 1.2 showed a sensitivity of 85% [95% CI 58–97%] and a specificity of 77% [95% CI 64–85%]. Similar results were observed when patients with DC were excluded.

### ICPW and outcome

**Fig. 3:**
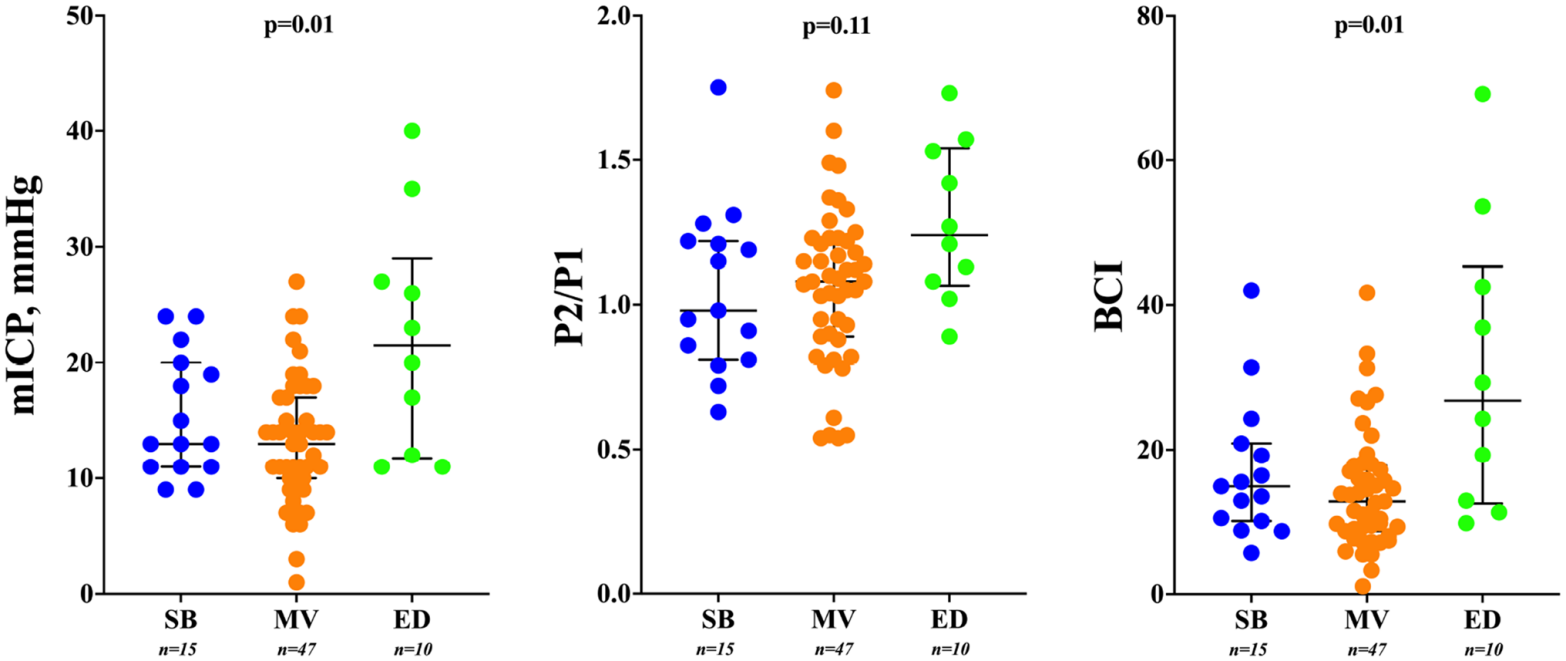
Distribution of mean intracranial pressure (mICP), P2/P1 ratio and brain compliance index (BCI), according to early outcomes *SB* spontaneous breathing, *MV* mechanical ventilation, *ED* early death)

**Fig. 4:**
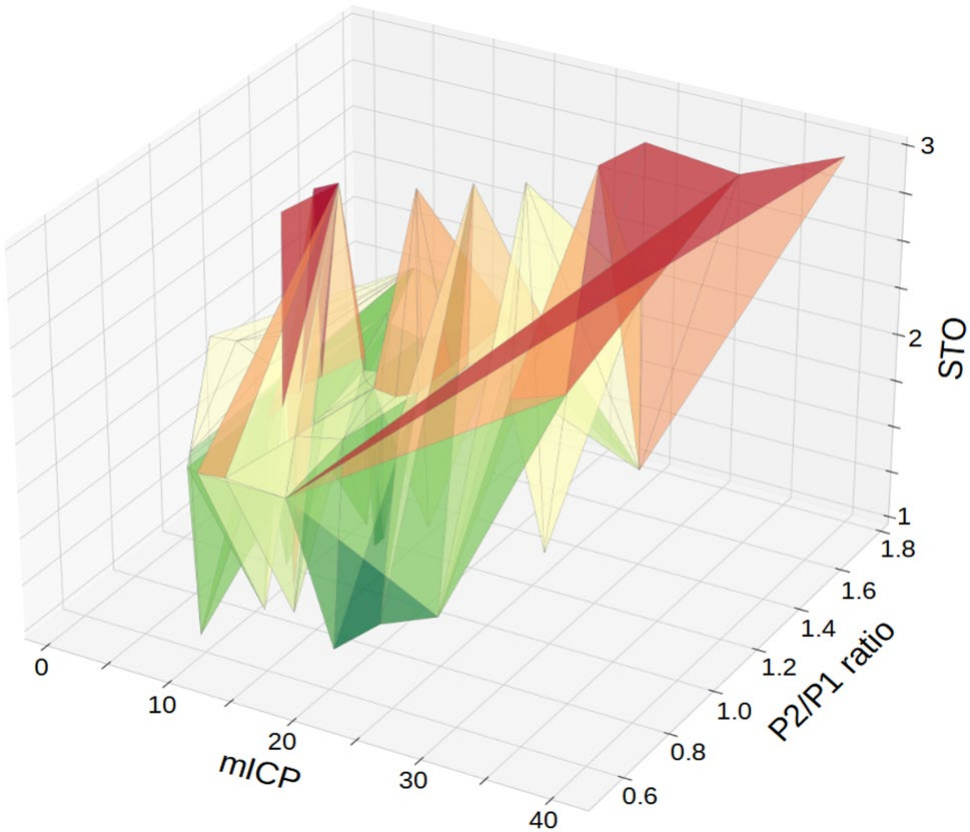
Tridimensional graphical depiction of the interaction between mean intracranial pressure (mICP), P2/P1 ratio and short-term outcomes (STO). Higher ICP levels combined with higher P2/P1 ratio results were observed for patients with poorer STO (red zone), whereas even borderline ICP levels, if associated with P2/P1 ratio under 1.2 were compatible often with favorable STO (green zone). Noteworthy, borderline mICP values combined with elevated P2/P1 ratio were frequently found for patients that remained under mechanical ventilation (MV, yellow-orange zone). STO 1- spontaneous breathing, 2- MV and 3- death. Electronic color enhancement is progressive according to the number of events observed for each particular value. Color is from green to red from better to poorer STO

mICP and the proportion of patients with mICP > 20 mmHg were significantly higher in the ED group when compared to the others (Table 1; Fig. 2). mICP and BCI were significantly higher for ED group compared to groups SB and MV. The AUROC of P2/P1 to predict ED was 0.71 [95% CI 0.53–0.87], and a P2/P1 cut-off of > 1.2 showed a sensitivity of 60% [95% CI 31–83%] and a specificity of 69% [95% CI 57–79%]. The AUROC of BCI to predict ED was 0.78 [95% CI 0.61–0.94], with a BCI > 19.3 showing a sensitivity of 70% [95% CI 40–89%] and a specificity of 79% [95% CI 67–87%]. Similar results were observed when patients with DC were excluded. Figures 3 and 4 depicts the interaction of mICP, P2/P1 ratio and STO. Higher mICP combined with higher P2/P1 ratios was associated with ED, interestingly, even borderline mICP values (between 18 and 22 mmHg) if associated with P2/P1 ratios under 1 were observed more often for the SB group. On the other hand, the same borderline mICP values if associated with P2/P1 ratios between 1.2 and 1.6 were observed more often for the MV group.

## Discussion

In this study, mICP values were significantly correlated with P2/P1 derived from NICPW analysis in a heterogeneous population of brain-injured patients. Moreover, P2/P1 values had, as for elevated mICP, prognostic value for ED. The presence of DC did not influence overall results. Hence, we report additional findings to the previous studies, from de Moraes et al. [18] which evaluated a cohort of 18 patients suffering from spontaneous intracranial hemorrhages without surgical procedures apart from the ICP catheter implant, and Brasil et al. [19] which performed a cross-sectional study of 41 ABI patients, mostly traumatic, with and without neurosurgical procedures, including DC. The primary endpoint for both studies, despite different populations and designs, was to correlate the invasive ICP and the parameters from the invasive ICP waveform, such as the P2/P1 ratio and time to peak with these same variables obtained noninvasively.

In experimental and clinical settings, the noninvasive P2/P1 ratio has been well correlated with ICP or applied as additional information to predict outcomes and assess shunting malfunctioning in children with hydrocephalus [24, 25] and idiopathic intracranial hypertension [26]. In COVID-19 patients, the combination of P2/P1 with TCD allowed to identify disturbances in cerebral hemodynamics (CH) and predict early poor outcome [27]. Moreover, the application of this system in previously unexplored health conditions has suggested interesting alterations in ICC among patients with end-stage renal disease under hemodialysis [28] and robotic prostatectomy surgery because of Trendelenburg positioning [29].

The clinical interest of P2/P1 should be on the understanding of the brain tolerance to different ICP values; as an example, if a patient with an ICP of 18 mmHg could be considered as having mICP values within acceptable ranges, the concomitant presence of P2/P1 > 1.2 might suggest poor ICC, potentially requiring additional investigations and interventions. On the opposite, an mICP of 23 mmHg, which should deserve prompt therapy according to guidelines, could be further evaluated in its pathogenesis (i.e., hyperthermia, awakening, fever) if P2/P1 ratio remains within normal ranges. A prospective study including additional neuromonitoring tools to better understand cerebral physiology during ICP surges is required to respond to this hypothesis and may fulfill the gaps left by numerical thresholds [30].

The classic studies of Marmarou et al. [17] and Langfitt et al. [31], by means of invasive ICP measurement, observed the relation between intracranial volume and ICP variations [32], whereas Nucci et al. [21] confirmed that changes in ICPW followed ICP variations; in particular, elevation of the second ICP peak was related to impaired ICC, although quantitative relations between ICPW peaks were not demonstrated in that study. Kazimierska et al. [33] performed intracranial elastance assessment by means of infusion test in normal pressure hydrocephalus patients and compared three techniques, including changes in P1 and P2 amplitudes, indicating that the ratio obtained from these peaks has good correlation with the intracranial volumetric manipulation.

ICPW is a well-known parameter for intensivists and neurosurgeons; however, clinical applicability of ICPW remains difficult because invasive systems do not routinely analyze P2/P1, being a clear recognition of the two peaks not always possible. The expansion of multimodal neuromonitoring could help to further understand how ICP values should be optimized in clinical practice. In particular, as the combination of ICP values with brain oxygenation [34, 35], there is potential to combine information from ICP invasive measurement and the NICPW analysis to better understand ICC after an ABI. Likewise, the combination of different noninvasive techniques also seems to improve ICC monitoring and outcome prediction [36].

This study has several limitations to acknowledge. First, a single session monitoring of these variables is clearly narrow for the outcome determination since ICP and ICC are dynamic properties that will vary continuously. Therefore, the association of P2/P1 with early mortality does not imply that altered ICC could be the only explanation for death, rather, this could be explained by the neurological damage itself even considering that our three groups disclosed no overall severity admission differences. Indeed, a cohort design is more adequate to outcome assessment, justifying a wider follow-up and register of IHT events that a patient may present during hospitalization. Second, a single-center study might influence patients’ management and early outcome, so that generalizability of overall results could be biased. Third, our results need for external validation. Although DC did not appear to significantly influence the applicability of NICPW analysis, some caution should be considered in such patients, who will require dedicated investigations with such monitoring tools.

## Conclusion

The novel noninvasive system can analyze biometric parameters extracted from the ICPW parameters obtained from cyclic spontaneous cranial deformation, which are correlated with ICP. These parameters seem to be adjuvants for intracranial compliance monitoring and may participate on the outcomes of acute brain injured patients.

## Electronic supplementary material

Below is the link to the electronic supplementary material.

Supplementary Material 1

Supplementary Material 2

Supplementary Material 3
