# Supplementary figures and images for: Noninvasive intracranial pressure waveforms for estimation of intracranial hypertension and outcome prediction in acute brain-injured patients
Source: J Clin Monit Comput. 2022 Nov 18;37(3):753–60. doi: 10.1007/s10877-022-00941-y (PMC9673225; doi:10.1007/s10877-022-00941-y)

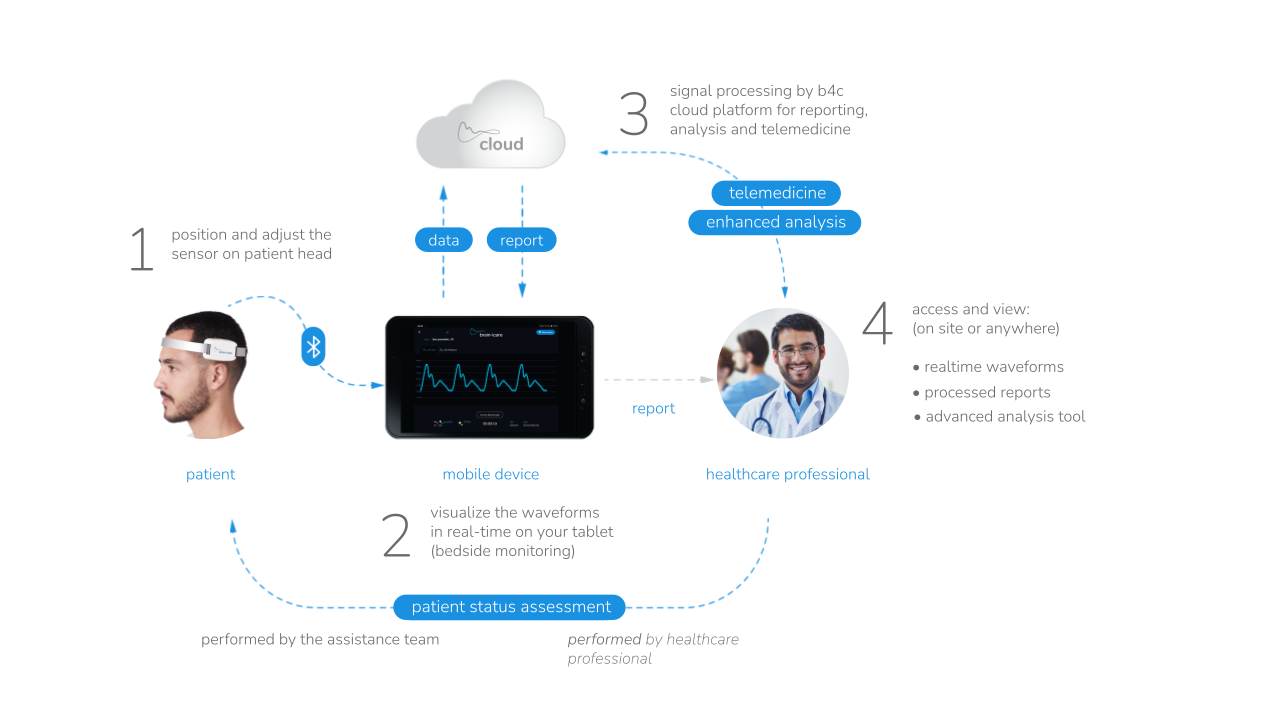


Figure 1. The current B4C system (with courtesy of brain4care corp).

Supplement: Supplementary file 2 — Supplementary Material 2 [file 10877_2022_941_MOESM2_ESM.docx]
