# Supplementary material for: Noninvasive intracranial pressure waveforms for estimation of intracranial hypertension and outcome prediction in acute brain-injured patients
Source: J Clin Monit Comput. 2022 Nov 18;37(3):753–60. doi: 10.1007/s10877-022-00941-y (PMC9673225; doi:10.1007/s10877-022-00941-y)
Supplement: Supplementary file 3 — Supplementary Material 3 [file 10877_2022_941_MOESM3_ESM.docx]

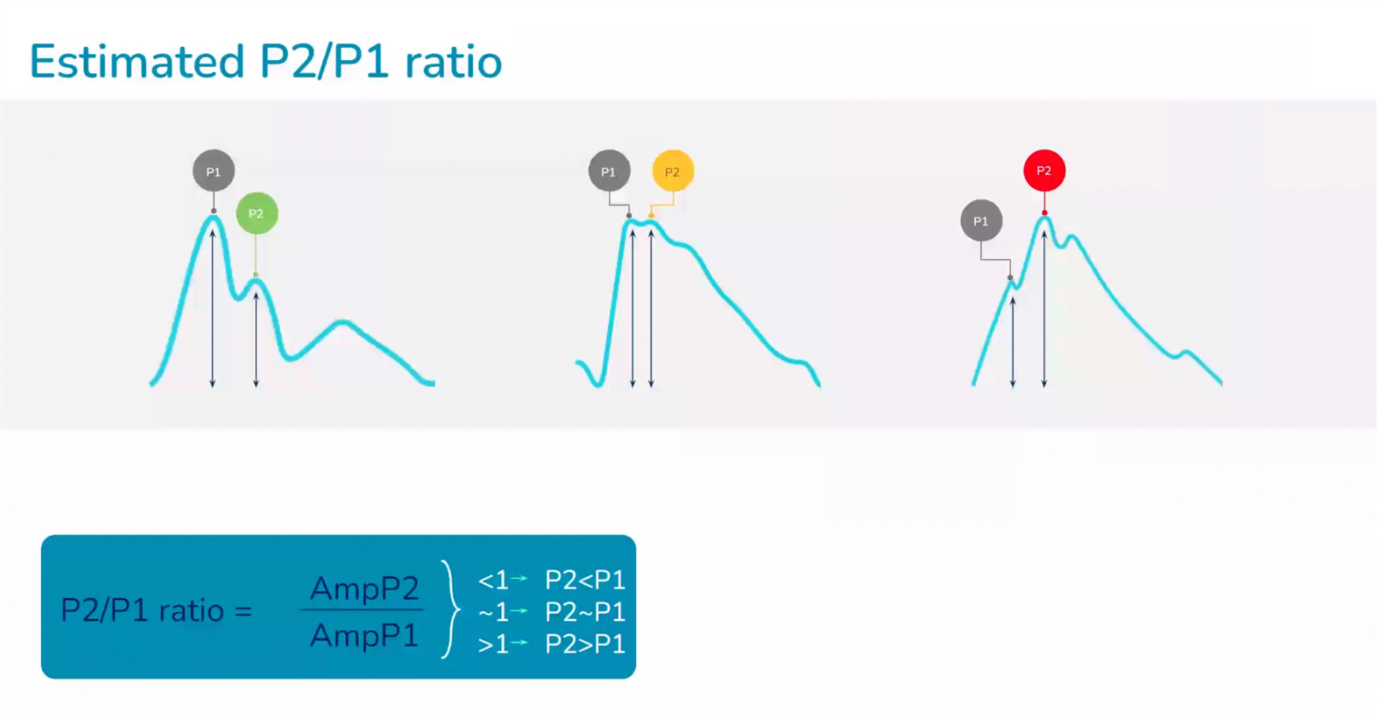


Supplemental figure. ICP waveform derived from each cardiac cycle depicts three different peaks. P1 represents cardiac/arterial upstroke, P2 is the tidal wave produced by blood spreading thru the brain, whereas P3 (not signaled here but is the deflection seen after P2) is the aortic valve closure, followed by diastole.
